# The association between mindfulness and social anxiety in university students: the chain mediation role of rumination and self-compassion

**DOI:** 10.3389/fpsyg.2026.1773864

**Published:** 2026-04-23

**Authors:** Lufang Cai, Ang Li

**Affiliations:** 1 Teacher Development College, Shaanxi Normal University, Xi’an, Shaanxi, China; 2 Department of Organization and Personnel, Zhengzhou Vocational College of Finance and Taxation, Zhengzhou, Henan, China; 3 School of Humanities and Design, Henan Open University, Zhengzhou, Henan, China

**Keywords:** mindfulness, rumination, self-compassion, social anxiety, university students

## Abstract

**Background:**

This study examined the association between mindfulness and social anxiety among university students and tested the chain mediating roles of self-compassion and rumination.

**Methods:**

Using random sampling and online surveys, 860 university students (465 males and 395 females) completed self-report measures of mindfulness, social anxiety, rumination, and self-compassion. Structural equation modeling was used to test the proposed relationships.

**Results:**

Mindfulness was significantly associated with lower social anxiety. Self-compassion and rumination mediated this relationship. Specifically, greater mindfulness was associated with higher self-compassion, which was in turn associated with lower rumination and, ultimately, lower social anxiety.

**Discussion:**

These findings suggest that mindfulness-based interventions may help reduce social anxiety by enhancing self-compassion and decreasing rumination. The results provide practical implications for educators and mental health professionals. Future research could further examine the long-term effects of these mediating mechanisms across different populations.

## 1 Introduction

University life represents a pivotal phase of socialization, where students encounter academic pressure and career choices alongside the challenges of complex and changing interpersonal relationships and emotional needs. As identity gradually develops and the process of social adaptation advances, individuals at this stage often exhibit heightened interpersonal sensitivity, which is linked to a relatively high prevalence of social anxiety (Wang et al., 2024; Xu and Li, 2024). Social anxiety refers to the fear experienced in social engagements when individuals concerns about being noticed, evaluated, or excluded by others (Xia et al., 2023). Such emotional responses may lead to avoidance behaviors, affecting classroom performance, interpersonal relationships, and ultimately reducing the quality of university life and future career prospects (Mou et al., 2024). More importantly, social anxiety has a strong connection with other psychological disorders, including depression and generalized anxiety disorder, carrying a risk of comorbidity. Without timely intervention, it may exacerbate individuals’ psychological distress (Bai et al., 2024; Cao et al., 2025). Therefore, systematically examining the mechanisms and determinants of social anxiety among university students holds significant theoretical and practical value for developing targeted psychological interventions and promoting their mental well-being.

### 1.1 Theoretical framework

Self-compassion theory (Neff, 2003b) proposes that when individuals face stress or adversity, they can alleviate negative emotions and promote mental health by approaching themselves with care, understanding, and a non-judgmental attitude, while acknowledging that people frequently feel misery. The theory defines self-compassion as a good self-attitude that consists of three fundamental elements: self-kindness, common humanity, and mindfulness. Among them, mindfulness is not only an essential part of self-compassion but functions as a safeguard when individuals cope with external stressors. This study examines mindfulness as measured by the MAAS scale, which measures a person’s awareness of right now, characterized by openness and acceptance to their ideas, feelings, and bodily sensations (Chu et al., 2025). Mindfulness within self-compassion, on the other hand, focuses more on accepting negative emotions through a non-judgmental attitude, particularly when facing personal suffering (Jiang et al., 2025). Although the two are related, they differ in psychological function. Mindfulness within self-compassion serves as an emotional regulation tool, aimed at helping individuals regulate their emotions, whereas the mindfulness referred to in this study focuses more on overall awareness of the present moment (Chu et al., 2025; Neff, 2003b). Both are psychological resources that enhance emotional well-being and psychological resilience, but they can be distinguished in their roles in emotional regulation and general awareness.

The core concept of mindfulness is to focus on the present moment and accept oneself. This concept not only aligns with the modern psychological understanding of mindfulness but also bears resemblance to philosophical ideas from history, such as Stoicism. Stoicism emphasizes accepting what cannot be controlled and concentrating on the here and now to achieve inner peace and happiness (Gomez et al., 2022). The commonality between the two lies in their shared advocacy for accepting life in the present moment with acceptance attitude, reducing worries about the past and future (Gomez et al., 2022; Neff, 2003b). Therefore, mindfulness is not only a technique for coping with stress and challenges but also a life philosophy that helps cultivate a calm mindset. The effectiveness of mindfulness in psychological interventions stems from its ability to help individuals reduce emotional distress and promote mental health.

Meanwhile, response style theory (Nolen-Hoeksema, 1991) emphasizes that the way individuals respond to negative emotions is pivotal in determining the trajectory of their emotional distress. Rumination, as a maladaptive cognitive processing style, can cause individuals to remain trapped in self-denial and anxious experiences after negative social encounters, thereby intensifying emotional distress.

Self-compassion theory and response style theory each provide important perspectives on how individuals cope with negative emotions. Self-compassion theory emphasizes enhancing emotional acceptance and regulation through self-kindness, common humanity, and mindfulness, thereby boosting psychological resilience (Neff, 2003b). In contrast, response style theory focuses on individuals’ cognitive approaches to negative emotions, particularly how rumination can exacerbate emotional distress (Nolen-Hoeksema, 1991). Although these two theories each explain the mechanisms of emotional regulation and emotional distress, they are complementary in their understanding of psychological processes. Self-compassion helps individuals accept negative emotions by providing a non-judgmental attitude and emotional regulation strategies, thus preventing them from falling into negative thought patterns of rumination and self-criticism, which in turn reduces the occurrence of rumination (Petwal et al., 2023). Response style theory, on the other hand, helps us understand how individuals can alleviate emotional distress by adjusting their thinking patterns, such as reducing rumination. By integrating these two theories, a more comprehensive explanation can be provided of how mindfulness alleviates social anxiety by enhancing self-compassion and reducing rumination, thereby revealing the pathways through which mindfulness contributes to emotional regulation and psychological adaptation.

### 1.2 Mindfulness and social anxiety

Mindfulness denotes the awareness and attention focused on the here and now, with openness and acceptance of one’s thoughts, feelings, and bodily experiences (Chu et al., 2025). In this study, mindfulness is viewed as a trait-like cognitive state that participants already possess, rather than as a therapeutic intervention. This state of awareness encourages individuals to fully accept their current experiences, refrain from overthinking past events or anticipating future concerns, and is associated with reduced social anxiety (Abarkar et al., 2023). Studies have consistently found a strong inverse association between mindfulness and social anxiety (Noda et al., 2022b). People who practice mindfulness are able to embrace emotional event with an openness attitude, reducing excessive concerns about others’ evaluations. This, in turn, helps them remain calm in social situations and alleviates anxiety (Li et al., 2023). As an illustration, Obuca et al. (2024) revealed that higher mindfulness was associated with lower perceived social anxiety. In addition, mindfulness can further alleviate anxiety by improving physiological responses associated with social anxiety, such as increased heart rate and sweating. It enhances individuals’ awareness of bodily sensations, allowing them to promptly identify the physiological indicators of anxiety and regulate them through relaxation techniques (Clear et al., 2020; Noda et al., 2023). Such improvements in physiological responses not only reduce anxiety but also mitigate the broader negative effects of social anxiety. In summary, mindfulness can help reduce social anxiety, enabling individuals to maintain psychological balance in social situations, minimize the interference of anxious emotions, and promote both mental well-being and social adjustment.

### 1.3 The mediating role of self-compassion

Self-compassion is an inward-directed form of compassion that reflects how individuals treat themselves when facing adversity or failure (Makadi and Koszycki, 2020). Studies have revealed a clear negative link between self-compassion and social anxiety (Holas et al., 2021). People with higher self-compassion often meet social pressure or negative emotions with kindness and acceptance, avoiding self-criticism and disengaging from persistent negative feelings. This approach helps regulate emotions and reduce social anxiety (Hytman and Kocovski, 2023). In addition, self-compassion helps individuals accept their imperfections, reduce excessive self-criticism, and adopt a more tolerant attitude toward themselves, thereby further lowering social anxiety (Gao et al., 2023). Self-compassion also enhances individuals’ confidence, allowing them to rely less on others’ evaluations to define their self-worth in social settings. Instead, they focus more on internal self-acceptance, which strengthens social confidence and reduces social anxiety (Santarelli et al., 2021). Therefore, self-compassion, by fostering healthy emotion regulation and enhancing social confidence, is closely linked to reduced social anxiety.

Building on this, mindfulness, as an important factor in promoting self-compassion, further enhances a person’s capacity to manage social anxiety. Studies have shown a significant positive link between mindfulness and self-compassion (Yu et al., 2020). For instance, Yang et al. (2022) observed that individuals with higher mindfulness levels typically show greater self-compassion. Mindfulness helps individuals to stay more engaged in the present moment and embrace various emotions and experiences with an openness and acceptance attitude, thereby laying the foundation for cultivating self-compassion (Bailey et al., 2023; Cheung and Djekou, 2024). In social difficulties, university students often fall into self-criticism, whereas those with higher mindfulness levels are less inclined to adopt self-critical thoughts. They are better equipped to embrace their flaws and imperfections, thereby enhancing self-compassion (Zhang and Shen, 2022). In addition, mindfulness can enhance individuals’ capacity for empathy, enabling them not only to understand others’ emotions but also to approach their own emotional distress with compassion and understanding, thereby further fostering self-compassion (Paucsik et al., 2021; Zhang et al., 2025). Although previous research indicate that mindfulness may enhance self-compassion, whether self-compassion mediates the link between mindfulness and social anxiety still requires further empirical examination. While existing literature provides preliminary evidence, systematic investigations of this mediating mechanism remain limited. Therefore, this study seeks to investigate the mediating role of self-compassion in the connection between mindfulness and social anxiety, thereby offering clearer guidance for both theoretical advancement and practical application.

### 1.4 The mediating role of rumination

Rumination is the repeated contemplation of negative life events and their outcomes without engaging in effective problem-solving (Wu et al., 2024). Studies have revealed a strong positive association between rumination and social anxiety (Xu and Li, 2024). For example, Wu et al. (2024), using a cross-lagged analysis of college students, discovered that rumination was linked to both their current social anxiety and their social anxiety 6 months later. In social situations, when university students experience negative events, rumination leads them to repeatedly recall these situations and remain immersed in negative emotions, thereby intensifying social anxiety (Kurtoğlu et al., 2024). In addition, when individuals become trapped in rumination, they often remain immersed in negative emotions and are unable to effectively employ coping strategies such as emotion shifting or relaxation techniques to alleviate anxiety (Zhang et al., 2024). Rumination can also intensify anticipatory anxiety about future social situations, leading individuals to overestimate potential threats or negative outcomes in social interactions and to experience excessive worry about upcoming encounters. This persistent anxious thinking makes it more difficult for individuals to cope with social situations calmly, further increasing social anxiety (Noda et al., 2022a; Shi et al., 2025). In summary, rumination traps individuals in a vicious cycle of anxiety from which they struggle to break free, greatly amplifying social anxiety levels.

In contrast, mindfulness plays a crucial role in alleviating rumination, providing a potential pathway to address the aforementioned vicious cycle. In addition, research has consistently shown a clear inverse association between mindfulness and rumination (Gu et al., 2022; Kim et al., 2022). Shi et al. (2023) reported that mindfulness can effectively reduce individuals’ rumination. People with greater mindfulness are more inclined to focus more on present experiences rather than repeatedly revisiting past negative events or excessively worrying about future situations, thereby reducing the occurrence of rumination (Bolzenkotter et al., 2025). In addition, mindfulness helps individuals recognize the onset of rumination and employ effective strategies to shift attention away from negative thoughts, while better managing negative emotions and avoiding excessive reflection, thereby further reducing rumination (de la Fuente-Anuncibay et al., 2021). Mindfulness can also reduce individuals’ negative cognitive biases, helping them accept negative emotions rather than viewing them as uncontrollable or permanent threats. By decreasing excessive reactions to negative emotions, mindfulness further reduces the occurrence of rumination (Gu et al., 2022). Overall, current evidence indicates that rumination is a major contributor to social anxiety, while mindfulness, serving as a valuable strategy for emotion regulation, can significantly reduce the occurrence of rumination. Therefore, exploring whether rumination mediates the link between mindfulness and social anxiety holds important theoretical and practical significance.

### 1.5 The chain mediation role of self-compassion and rumination

Studies have consistently demonstrated a clear negative relationship between self-compassion and rumination (Ogawa and Kojima, 2025). Those with greater self-compassion often employ adaptive emotion regulation strategies to disengage from negative emotions rather than repeatedly recalling negative events, thereby reducing rumination (Miyagawa and Taniguchi, 2022). In addition, individuals with high self-compassion can remain calm when facing social pressure, avoiding immersion in negative emotions and self-blame. In contrast, those lacking self-compassion may intensify rumination by focusing excessively on failures and negative events (Petwal et al., 2023). Jansen et al. (2021b) discovered that individuals with greater self-compassion showed reduced levels of rumination.

Mindfulness enhances individuals’ self-awareness and helps them accept themselves with an openness attitude, thereby fostering the development of self-compassion (Yu et al., 2020). Self-compassion theory suggests that people with greater self-compassion are more capable of accepting their imperfections when facing social pressure, rather than repeatedly recalling past failures or negative situations, thereby reducing the occurrence of rumination (Jansen et al., 2021a). Rumination is a form of negative cognitive response. According to response style theory, the way individuals respond to emotional distress directly is related to their mental health. A tendency toward rumination can exacerbate social anxiety, as individuals may become overly concerned about others’ evaluations and avoid social situations, thereby intensifying social anxiety (Zhang et al., 2024). As an illustration, Wu et al. (2025) indicated that mindfulness was related to university students’ social anxiety by enhancing self-compassion and reducing rumination (Xu and Li, 2024). Therefore, from the perspectives of self-compassion theory and response style theory, mindfulness may alleviate social anxiety via a chain mediation pathway involving self-compassion and rumination. Through this sequential mechanism, mindfulness not only directly facilitates emotion regulation but also indirectly helps students cope more effectively with social pressure and enhance mental wellness by encouraging self-compassion and reducing rumination.

### 1.6 The present study

**Figure 1:**
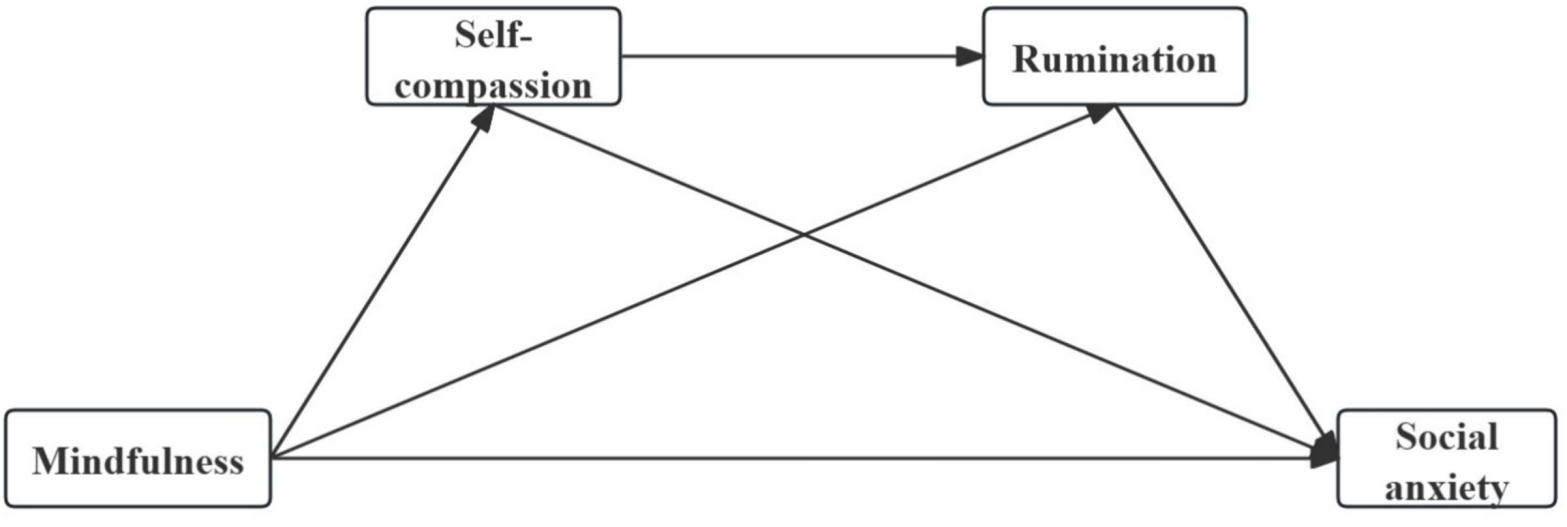
Conceptual model.

Although earlier research has investigated the link between mindfulness and social anxiety, its underlying psychological mechanisms have not been systematically explored. To date, no research has clearly determined whether mindfulness is related to social anxiety in university students by increasing self-compassion and decreasing rumination. To this end, this study innovatively integrates self-compassion theory and response style theory, proposing a chain mediation model to investigate how mindfulness is related to social anxiety through the sequential mediating roles of self-compassion and rumination. Self-compassion helps individuals cope with negative emotions with a gentle and non-judgmental attitude, preventing them from falling into self-criticism and reducing the occurrence of rumination (Neff, 2003b), thereby alleviating social anxiety. Unlike previous studies, this research is the first to systematically explore the psychological mechanism through which mindfulness is related to social anxiety via the chain mediation of self-compassion and rumination, filling a gap in the current literature. Through this novel model, the study provides fresh viewpoints and empirical support for psychological intervention techniques targeting university students. Figure 1 presents the conceptual model, and the corresponding hypotheses are put forth in accordance:

*H1*: Mindfulness shows a significantly negative association with social anxiety.

*H2*: Self-compassion mediates the connection between mindfulness and social anxiety.

*H3*: Rumination mediates the connection between mindfulness and social anxiety.

*H4*: Self-compassion and rumination sequentially mediate the connection between mindfulness and social anxiety.

## 2 Methods

### 2.1 Participants and procedure

The data was collected between June to July 2025. To enhance sample representativeness and minimize selection bias, we carried out a questionnaire-based survey with students drawn through random sampling from three universities in China. Prior to administering the formal survey, the research team informed the universities and the students about the study’s objectives and procedures. Subsequently, an online questionnaire link, developed using the Wenjuanxing platform,^1^ was shared through class WeChat groups. Students completed and submitted the questionnaire independently within the specified time frame. To assess whether the sample size was adequate, *a priori* power analysis was performed with G*Power 3.1 (Faul et al., 2009). Based on a medium effect size (*f*^2^ = 0.15), *α* = 0.05, and power = 0.95, the research showed that a minimum of 129 participants were required. With 925 individuals overall, the sample size was larger than needed, guaranteeing adequate statistical power. We excluded 65 invalid responses, which were identified as cases with more than 20% missing items or with over 80% of items marked as “strongly agree” or “strongly disagree.” As a result, 860 valid questionnaires were kept, resulting in a 92.97% effective response rate. Participants were primarily sophomores (42.8%) (see Table 1).

**Table 1:** Demographic characteristics.

Structure	Category	*N*	Percentage (%)
Gender	Male	465	54.1
	Female	395	45.9
Grade	Freshman	304	35.3
	Sophomore	368	42.8
	Junior	165	19.2
	Senior	23	2.7
Household registration	Rural	657	76.4
	Urban	203	23.6

### 2.2 Measures

#### 2.2.1 Mindfulness

This study measured mindfulness among university students using the Chinese version of the Mindful Attention Awareness Scale (MAAS) (Brown and Ryan, 2003), in suite Chinese version adapted by Deng et al. (2012). The test consists of 15 items scored on a 6-point scale. The Cronbach’s alpha was 0.944. Confirmatory factor analysis (CFA) indicated a satisfactory structural fit, with all indices meeting recommended standards (χ^2^/df = 2.830, CFI = 0.978, NFI = 0.966, IFI = 0.978, RMSEA = 0.046).

#### 2.2.2 Rumination

This study assessed rumination among university students using the Ruminative Responses Scale (Nolen-Hoeksema and Morrow, 1991), in suite Chinese version adapted by Han and Yang (2009). The instrument includes 22 items covering three dimensions: symptom rumination, compulsive thinking, and reflective pondering. It uses a 4-point scale. The Cronbach’s alpha was 0.953. CFA indicated a good structural fit, with all indices meeting recommended standards (*χ*^2^/df = 2.148, CFI = 0.976, NFI = 0.956, IFI = 0.976, RMSEA = 0.037).

#### 2.2.3 Self-compassion

This study assessed self-compassion among university students using the Self-Compassion Scale (Neff, 2003a), in suite Chinese version adapted by Chen et al. (2011). The instrument includes 12 items covering three dimensions: self-kindness, mindfulness, and common humanity. It uses a 5-point Likert scale. The Cronbach’s alpha was 0.920. CFA showed a satisfactory structural fit, with all indices meeting recommended standards (*χ*^2^/df = 4.197, CFI = 0.967, NFI = 0.958, IFI = 0.968, RMSEA = 0.061).

#### 2.2.4 Social anxiety

This study measured social anxiety among university students using the Social Anxiety subscale from the Self-Consciousness Scale (Fenigstein et al., 1975). The instrument includes six items on a 5-point Likert scale. The Cronbach’s alpha was 0.869. CFA showed a satisfactory structural fit, with all indices meeting recommended standards (*χ*^2^/df = 4.136, CFI = 0.987, NFI = 0.983, IFI = 0.987, RMSEA = 0.060).

### 2.3 Data analysis

We used Smart PLS 4.0 for data analysis. This software was selected because it maximizes the explanatory power of endogenous latent variables when handling non-normally distributed data, moderate sample sizes, and complex models (Hair et al., 2022). This study sought to investigate the link between mindfulness and social anxiety in university students and to test the chain mediation effects of self-compassion and rumination. The model included four latent variables, 55 measuring items, and 860 valid responses, making it relatively complex in structure. In addition, all variables had *p* < 0.001 according to the Shapiro–Wilk test results, indicating that the data deviated from a normal distribution. However, Smart PLS-SEM is known for its robustness to non-normal data distributions. Therefore, Smart PLS was used as an appropriate and efficient analytical tool.

## 3 Results

### 3.1 Descriptive statistics and correlation analysis

According to correlation analysis, mindfulness was strongly positively correlated with self-compassion and negatively correlated with rumination and social anxiety (Table 2).

**Table 2:** Descriptive statistics and correlation analysis.

Variable	M ± SD	Sk	Kur	Mindfulness	SC	Rumination	SA
Mindfulness	4.14 ± 1.11	−0.83	−0.14	1			
SC	3.18 ± 0.78	−0.11	−0.48	0.60***	1		
Rumination	2.17 ± 0.65	1.34	0.56	−0.61***	−0.61***	1	
SA	3.05 ± 0.82	0.18	0.22	−0.65***	−0.64***	0.63***	1

SC, self-compassion; SA, social anxiety; ***p < 0.001.

### 3.2 Common method Bias (CMB)

This study used Harman’s single-factor test to do a CMB test. Four factors with eigenvalues greater than 1 were found in the exploratory factor analysis results. The first component explained 38.931% of the variance, which is less than the threshold of 40% (Podsakoff and Organ, 1986). This suggests that the study’s data do not contain any significant CMB.

### 3.3 SEM analysis

#### 3.3.1 CFA

This study conducted confirmatory factor analysis on the overall measurement model, which included four variables: mindfulness, self-compassion, rumination, and social anxiety. CFA showed that the observed variables had factor loadings between 0.56 to 0.81, all above the 0.50 threshold. This step ensured that the constructs were adequately represented by the measurement items before conducting the PLS-SEM analysis. The results of the CFA were consistent with the requirements of PLS-SEM, verifying the measurement model’s validity (see Table 3) (Ji et al., 2024).

**Table 3:** Model fit indices.

Fit index	Reference	Model
CMIN/DF	<5	1.87
RMSEA	<0.08	0.03
SRMR		0.04
GFI	>0.85	0.89
AGFI		0.88
CFI		0.95
IFI		0.95
TLI		0.95

#### 3.3.2 Measurement model

Composite reliability (CR) and item factor loadings were used to evaluate reliability. In general, item loadings should exceed 0.70 (Hair et al., 2021). Although some items in this study had outer loadings below this threshold, they were retained as their CR or average variance extracted (AVE) were within the desirable range. Internal consistency reliability was measured using CR, with the criterion that values should be above 0.70 (Hair et al., 2021). The item loadings ranged from 0.610 to 0.820, and the CR values ranged from 0.902 to 0.957, indicated strong reliability.

Validity assessment included convergent validity and discriminant validity. Convergent validity was examined using the AVE, and the results showed that all AVE values ranged from 0.502 to 0.605, exceeded the 0.50 threshold (Hair et al., 2021). Discriminant validity was assessed using the heterotrait-monotrait ratio (HTMT) and the traditional Fornell–Larcker criterion (Hair et al., 2021). All HTMT values (see Table 4) were below the 0.90 threshold. At the same time, the Fornell–Larcker criterion was satisfied because all of the correlations between constructs were all smaller than the square root of their respective AVE (see Table 5) (Hair et al., 2021). Therefore, results from both the HTMT and Fornell-Larcker assessments confirmed that all constructs in this study demonstrated high discriminant validity.

**Table 4:** HTMT criterion.

Variable	Mindfulness	Rumination	SA	SC
Mindfulness				
Rumination	0.637			
SA	0.716	0.688		
SC	0.648	0.656	0.720	

**Table 5:** Fornell-Larcker criterion.

Variable	Mindfulness	Rumination	SA	SC
Mindfulness	***0.748***			
Rumination	−0.606	***0.708***		
SA	−0.651	0.629	***0.778***	
SC	0.605	−0.618	−0.648	***0.732***

The diagonal values in bold and italics represent the square root of AVE values.

#### 3.3.3 Structural model

We evaluated the structural model analysis’s explanatory capacity and dependability using metrics including path coefficients, coefficients of determination (R^2^), and collinearity diagnostics. We assessed multicollinearity among the latent variables using collinearity diagnostics (Hair et al., 2021). According to conventional criteria, the variance inflation factor (VIF) should remain below 3.3, and meeting this standard shows that severe multicollinearity is not present. The model had no significant multicollinearity issues (Table 6).

**Table 6:** VIF values.

Variable	Mindfulness	Rumination	SA	SC
Mindfulness		1.579	1.829	1.000
Rumination			1.875	
SA				
SC		1.579	1.874	

**Figure 2:**
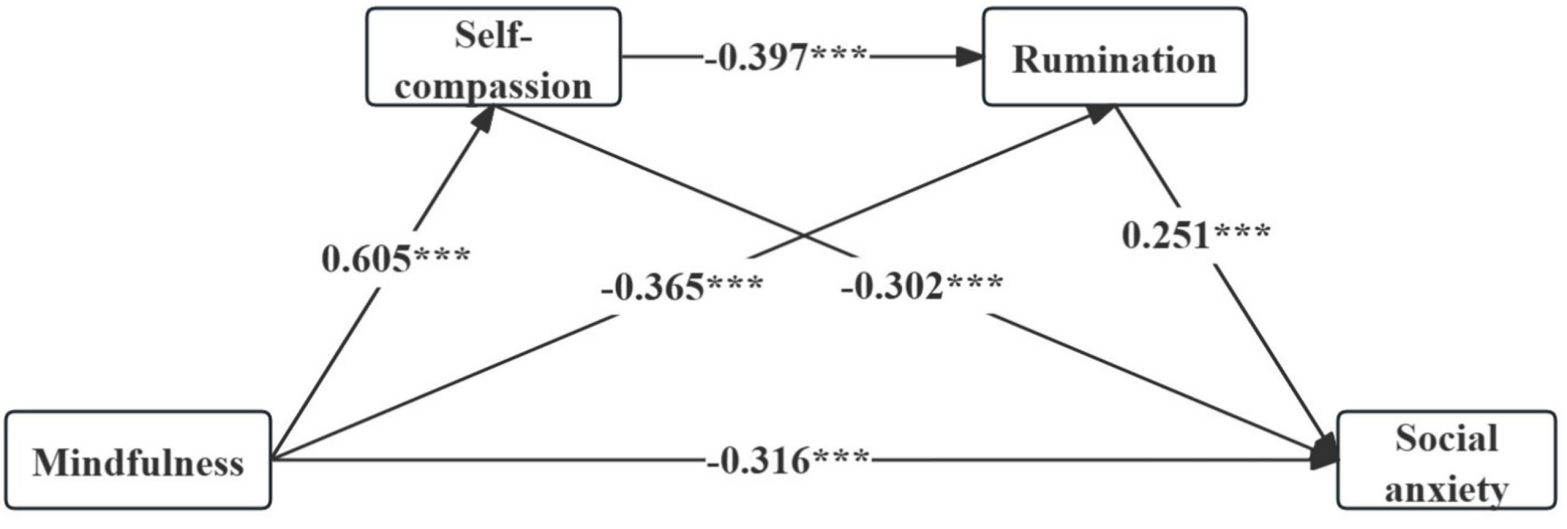
Path diagram.

We used a PLS analysis with 5,000 bootstrap resamples to examine the size and statistical significance of the model’s path coefficients. The significance tests in the structural model examined how strongly the exogenous variables related to the endogenous variables. Results indicated that mindfulness (*β* = −0.316, *t* = 9.184, *p* < 0.001) was significantly associated with social anxiety (Table 7; Figure 2).

**Table 7:** The chained mediation models of SC and rumination in the link between mindfulness and SA (*n* = 860).

Path	*β*	*t*	*p*	Result
Mindfulness → Rumination	−0.365	8.875	<0.001	Significant
Mindfulness → SA	−0.316	9.184	<0.001	Significant
Mindfulness → SC	0.605	24.639	<0.001	Significant
Rumination → SA	0.251	7.818	<0.001	Significant
SC → Rumination	−0.397	10.284	<0.001	Significant
SC → SA	−0.302	9.063	<0.001	Significant

Table 8 shows that the *R*^2^ results indicate strong explanatory capability, with the predictors explaining 55.9% of the variance in social anxiety. In addition, all Q^2^ values exceeded zero, suggesting that the model possessed satisfactory predictive relevance (Hair et al., 2021).

**Table 8:** Explanatory power and predictive relevance.

Variable	R^2^	Q^2^	Model fit
Rumination	0.467	0.231	
SA	0.559	0.331	SRMR:0.039
SC	0.367	0.191	NFI:0.900

### 3.4 Mediation analysis

We used the bootstrapping approach with 5,000 subsamples to estimate both indirect and direct effects (Nitzl et al., 2016). We adopted this approach to evaluate the significance and characterize the nature of the mediation effects. The analysis findings indicated that the chain mediation effect of self-compassion and rumination was significant (see Table 9).

**Table 9:** Mediation effects of rumination and SC.

Path	Indirect effect	*t*	*p*	Direct effect	*t*	*p*	Mediation type
Mindfulness → SC → SA	−0.183	8.398	<0.001	−0.316	9.184	<0.001	CPM
Mindfulness → Rumination → SA	−0.092	5.479	<0.001	−0.316	9.184	<0.001	CPM
Mindfulness → SC → Rumination → SA	−0.060	6.456	<0.001	−0.316	9.184	<0.001	CPM

CPM, complementary partial mediation.

## 4 Discussion

Based on self-compassion theory and response style theory, this study aims to explore the relationship between mindfulness, self-compassion, rumination, and social anxiety among university students, as well as to analyze the chain mediation effect of self-compassion and rumination. The findings indicate a strong negative link between mindfulness and social anxiety, with self-compassion and rumination serving as complementary partial mediators in the link between mindfulness and social anxiety. Additionally, self-compassion and rumination were also important chain mediators in this approach. The SEM analysis results confirmed all hypotheses, with these constructs explaining 55.9% of the variance in social anxiety. This discovery offers a fresh theoretical framework for comprehending how university students can alleviate social anxiety.

The results demonstrating a strong and negative link between mindfulness and social anxiety, supporting H1. This finding aligns with Li et al. (2023), suggesting that mindfulness, as a protective psychological resource, is associated with reduced social anxiety. Mindfulness can reduce excessive self-focus, encouraging individuals to view their social performance with an openness attitude. This approach helps lower self-criticism and excessive monitoring of self-image, thereby alleviating social anxiety (Obuca et al., 2024). In addition, mindfulness helps university students focus more on present experiences, reducing anticipatory concerns about negative evaluations and excessive worry about others’ judgments, thereby further alleviating social anxiety (Noda et al., 2022b). Individuals with higher levels of mindfulness tend to exhibit greater awareness, which helps them recognize early signs of physical tension in social situations. By detecting these early signals, individuals are better able to apply strategies, such as breath regulation, to manage physiological arousal, thereby helping to mitigate anxiety during social interactions (Noda et al., 2023). In summary, mindfulness is linked to reduced anxiety levels in social situations among university students.

The findings indicate that self-compassion mediated the link between mindfulness and social anxiety, supporting H2. This finding aligns with Cheung and Djekou (2024), showing that mindfulness enhances self-compassion in university students, which is linked to reduced anxiety in social situations (Hytman and Kocovski, 2023). This finding also supports self-compassion theory, which posits that mindfulness encourages individuals to face their flaws and difficulties with an openness attitude. People with higher mindfulness levels often embrace themselves more fully, thereby exhibiting greater self-compassion (Neff, 2003b; Zhang and Shen, 2022). Self-compassion, as a positive resource for emotion regulation, can help reduce negative emotional responses in social contexts (Holas et al., 2021). University students with greater self-compassion are more tolerant of their imperfect performance when facing social evaluative pressure, viewing social failures or awkward situations as normal experiences. This perspective reduces feelings of shame and excessive worry about negative evaluations, thereby easing social anxiety (Santarelli et al., 2021). Therefore, mindfulness alleviates anxiety experiences by enhancing individuals’ self-compassion, providing them with a positive psychological resource to cope with social challenges.

The results indicate that rumination mediated the link between mindfulness and social anxiety, supporting H3. This finding aligns with Kim et al. (2022), indicating that university students with higher mindfulness are less prone to engage in rumination and tend to report lower social anxiety (Kurtoğlu et al., 2024). A mindfulness tendency enhances individuals’ awareness and acceptance toward present experiences, reducing repeated thoughts about negative social events. This helps prevent emotionally driven repetitive thinking, thereby decreasing the occurrence of rumination (Kim et al., 2022). Individuals who engage in less rumination tend to remain more composed in social situations and exhibit lower social anxiety (Zhang et al., 2024). However, when individuals fall into rumination, they often magnify their subjective perception of social threats and repeatedly review their performance after social interactions, anticipating others’ evaluations. This process intensifies feelings of threat and shame in social contexts, thereby increasing the level of social anxiety (Xu and Li, 2024). Therefore, mindfulness can indirectly ease university students’ tension and avoidance behaviors in social settings by inhibiting rumination.

The results show that self-compassion and rumination sequentially mediated the link between mindfulness and social anxiety, supporting H4. This result aligns with Wu et al. (2025), suggesting that mindfulness fosters greater self-compassion, which in turn reduces their tendency to ruminate, ultimately helping to alleviate social anxiety (Xu and Li, 2024). When individuals have higher mindfulness, they are more inclined to view their shortcomings and discomfort in social situations with a gentle, accepting attitude, thereby enhancing self-compassion (Yang et al., 2022). Those with high self-compassion inclined to accept their emotions after experiencing negative events, which reduces repeated reflection on past incidents and, in turn, lowers rumination levels (Petwal et al., 2023). As a key cognitive mechanism that maintains and exacerbates social anxiety, rumination, when reduced, can effectively alleviate individuals’ tension and avoidance responses in social situations (Wu et al., 2024). Conversely, excessive rumination leads individuals to repeatedly replay mistakes or perceived negative evaluations from social situations in their minds, thereby intensifying social anxiety (Shi et al., 2025). Therefore, self-compassion and rumination form a chain mediation pathway, in the link between mindfulness and social anxiety: mindfulness enhances self-compassion, self-compassion reduces rumination, and the decrease in rumination ultimately lowers individuals’ negative emotional responses in social situations.

## 5 Implications

### 5.1 Theoretical implications

First, this study enriches the application of mindfulness in university students’ mental health and extends its theoretical scope to social anxiety contexts. Existing studies have looked at the link between mindfulness and general emotional disorders like depression and generalized anxiety, with relatively few studies addressing its regulatory mechanisms for anxiety responses in specific social situations. By empirically testing the link between mindfulness and social anxiety, this study provides new evidence supporting theoretical applicability of mindfulness in interventions targeting social emotional disorders.

Second, this study combines self-compassion theory and response style theory, introduces self-compassion and rumination as sequential mediators, helping to uncover the internal psychological mechanisms linking mindfulness and social anxiety and deepening the understanding of their relationships. Mindfulness, as a cognitive state, enhances an individual’s awareness of current emotions and thoughts, allowing them to accept these experiences in an openness way. This helps reduce reactions to negative emotions and break the cycle of negative emotional responses. Self-compassion, as a positive self-attitude, enables individuals to treat themselves with kindness, promoting emotional regulation and alleviating anxiety. At the same time, mindfulness effectively suppresses rumination, a negative thinking pattern that often exacerbates social anxiety. By reducing rumination, mindfulness alleviates the cognitive load of social anxiety and improves emotional states. By incorporating self-compassion and rumination into a chain mediation model, this study constructs a comprehensive pathway from mindfulness to cognitive processing and then to social anxiety, offering a more holistic perspective on the emotional regulation process.

Finally, in terms of methodology, this study employs a chain mediation model, responds to the research approach in the field of emotional regulation that focuses on the integration of multiple mechanisms. This model integrates self-compassion (a positive self-regulation resource) and rumination (a negative cognitive pattern) within the same framework, revealing their sequential mediating roles in the link between mindfulness and social anxiety. It provides a valuable analytical framework for upcoming studies on the core components and pathways of mindfulness interventions.

### 5.2 Practical implications

First, for university students, the findings suggest that mindfulness can alleviate social anxiety by fostering self-compassion and lowering rumination, providing a feasible pathway for psychological adjustment. Through regular mindfulness practices, such as focused breathing, and non-judgmental awareness of emotions, and body scans (Body scanning is a mindfulness technique that involves focusing on the sensations in different parts of the body), students can improve their awareness of emotional responses, reduce self-criticism and negative reflection, and strengthen their adaptability and psychological resilience in social contexts. These improvements can, in turn, help ease withdrawal and discomfort caused by anxiety over interpersonal evaluation.

Second, this study provides two specific recommendations for university mental health education and counseling services. On one hand, when designing and conducting mental health courses or group counseling, incorporating mindfulness training can help students enhance their emotional awareness and management skills. On the other hand, intervention programs can focus more on guiding students to cultivate self-compassion and reduce rumination, for example, by leading students through themed activities that encourage a more compassionate attitude toward their social performance, thereby interrupting the vicious cycle of negative thinking. This offers potential pathways for university mental health professionals to design targeted social anxiety prevention and intervention programs.

### 5.3 Limitations and future research directions

Although this study identified a chain mediation mechanism in which mindfulness affects social anxiety via self-compassion and rumination, several limitations remain and warrant further exploration and refinement in future research.

First, this study employed a cross-sectional survey approach, which restricts the ability to draw causal links between the variables. Although the chain mediation pathway was derived from the theoretical model, it cannot confirm the temporal sequence among mindfulness, self-compassion, rumination, and social anxiety. Therefore, the study results should be interpreted as reflecting associations consistent with the theoretical model, rather than as evidence of established causal pathways. Future research could use longitudinal tracking or experimental intervention designs to more clearly verify the causal pathways and dynamic changes among these variables.

Second, this study’s reliance on self-report questionnaires introduces potential biases, including social desirability effects and individual cognitive biases. To enhance the objectivity and diversity of the findings, future studies could integrate multiple data collection methods, including behavioral measures, interviews, and physiological markers, to improve the reliability and ecological validity of the conclusions.

Finally, this study examined Chinese university students. Although the sample has a certain degree of representativeness, its relatively homogeneous cultural background may constrain the external validity of the findings. In diverse cultural settings, individuals’ understanding and experience of mindfulness, self-compassion, and social interactions may vary, potentially affecting both the manifestation of social anxiety and its underlying mechanisms. Future research could adopt a cross-cultural perspective to compare relationship patterns among variables across different cultural contexts, expanding the generalizability of the findings and broadening the scope of theoretical explanations.

## 6 Conclusion

Drawing on self-compassion theory and response style theory, this study investigated how mindfulness relates to social anxiety among university students, highlighting the sequential mediating roles of self-compassion and rumination. The findings showed that mindfulness was linked to increased self-compassion and reduced rumination, with these two factors forming a significant sequential mediation pathway to reduced social anxiety levels. This finding broadens the application of self-compassion theory and response style theory to the domain of social emotional disorders and offers fresh insight into the psychological mechanisms of mindfulness in emotion regulation. The study results highlight the potential role of mindfulness as a cognitive state in alleviating social anxiety, particularly among university students, where its importance becomes more pronounced as psychological stress increases. Although the study provides preliminary evidence, it remains limited by its cross-sectional design and culturally homogeneous sample. Future research could further validate and refine the proposed mechanisms through longitudinal tracking and cross-cultural comparisons.

## Data Availability

The datasets presented in this study can be found in online repositories. The names of the repository/repositories and accession number(s) can be found in the article/supplementary material.

## Footnotes

1
https://www.wjx.cn/
